# Supplementary material for: Cinnamaldehyde Inhibits Postharvest Gray Mold on Pepper Fruits via Inhibiting Fungal Growth and Triggering Fruit Defense
Source: Foods. 2023 Sep 16;12(18):3458. doi: 10.3390/foods12183458 (PMC10530028; doi:10.3390/foods12183458)
Supplement: Supplementary file 1 [file foods-12-03458-s001.zip › foods-2571191-supplementary.pdf]

Supplementary Material

Cinnamaldehyde Inhibits Postharvest Gray Mold on Pepper Fruits via Inhibiting Fungal Growth and Triggering Fruit Defense

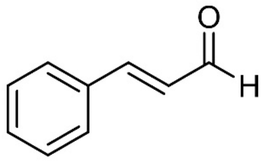

Figure S1. The chemical structure of cinnamaldehyde.

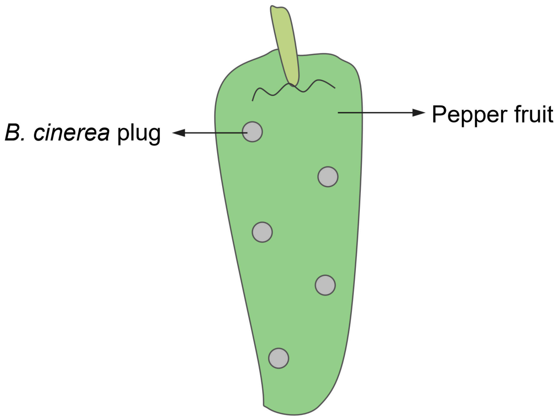

Figure S2. The schematic model for the inoculation of *B. cinerea* on the surface of pepper fruit. Gray circles represent the PDA-cultured *B. cinerea* mycelial plugs. Each fruit was inoculated with five mycelial plugs with even distribution on fruit surface.
